# Comprehensive analysis of miRNA profiling in *Schistosoma mekongi* across life cycle stages

**DOI:** 10.1038/s41598-024-52835-5

**Published:** 2024-01-29

**Authors:** Pavaret Sivapornnukul, Ariya Khamwut, Prangwalai Chanchaem, Phiraphol Chusongsang, Yupa Chusongsang, Paporn Poodeepiyasawat, Yanin Limpanont, Onrapak Reamtong, Sunchai Payungporn

**Affiliations:** 1 Center of Excellence in Systems Microbiology (CESM), Department of Biochemistry, Faculty of Medicine, Chulalongkorn University, Bangkok, 10330 Thailand; 2 Department of Social and Environmental Medicine, Faculty of Tropical Medicine, Mahidol University, Bangkok, 10400 Thailand; 3 Department of Molecular Tropical Medicine and Genetics, Faculty of Tropical Medicine, Mahidol University, Bangkok, 10400 Thailand

**Keywords:** Parasitology, Non-coding RNAs

## Abstract

*Schistosoma mekongi*, a significant schistosome parasite, has various life stages, including egg, cercaria, female, and male, that play crucial roles in the complex life cycle. This study aimed to explore the microRNA (miRNA) profiles across these developmental stages to understand their potential functions and evolutionary significance, which have not been studied. Pre-processed sequencing reads of small RNA (sRNA) were obtained, and annotations were performed against the *S. japonicum* reference miRNA database. Results indicated marked variations in miRNA profiles across different life stages, with notable similarities observed between female and male *S. mekongi*. Principal Coordinate Analysis (PCoA) and unsupervised clustering revealed distinct miRNA signatures for each stage. Gene ontology (GO) analysis unveiled the potential roles of these miRNAs in various biological processes. The differential expression of specific miRNAs was prominent across stages, suggesting their involvement in crucial developmental processes. Furthermore, orthologous miRNA analysis against various worm species revealed distinct presence–absence patterns, providing insights into the evolutionary relationships of these miRNAs. In conclusion, this comprehensive investigation into the miRNA profiles of *S. mekongi* offers valuable insights into the functional and evolutionary aspects of miRNAs in schistosome biology.

## Introduction

Schistosomes are trematode parasites responsible for schistosomiasis, a disease that afflicts millions worldwide^1^. The life cycle of schistosomes is intricate, involving various developmental stages and multiple hosts^2,3^. *S. mekongi*, endemic to the Mekong River basin, primarily affects populations in Laos and Cambodia, with an estimated 80,000 individuals at risk in specific regions such as Stung Treng and Kratie provinces^4–6^. The transmission is intricately linked to the intermediate host, *Neotricula aperta*, found in the river’s rocky banks, with a distinct seasonal transmission cycle observed during the dry months^4–6^. Clinically and notably, *S. mekongi* infections typically presents with lower egg counts compared to other species, less than 150 EPG in most cases^6–8^. In contrast to other schistosomes, such as *S. mansoni* and *S. japonicum*, acute symptoms (i.e., Katayama fever) and polypoid lesions in the colon are rarely observed in *S. mekongi* infections^6–8^. Hence, understanding the biology and molecular mechanisms underpinning the various stages of the schistosome life cycle is pivotal for developing effective strategies againt the schistosome.

MicroRNAs (miRNAs) involvement in modulating a wide range of biological processes, from development to disease progression, has been recognized in various organisms, including parasites^9^. Given the profound roles miRNAs play in biological processes, there has been growing interest in understanding their functions within parasitic organisms and potential applications as therapeutic targets^10,11^.

In schistosomes, miRNAs have been implicated in essential physiological processes, governing parasite development, maturation, and host interaction^10,12^. Previous studies have explored miRNA profiles in other schistosome species, such as *S. japonicum* and *S. mansoni*, providing valuable insights into their roles in parasite biology^13,14^. These findings have provided a clue on the molecular mechanisms underlying schistosome development and adaptation to diverse environments^13,14^. However, the miRNA landscape across the developmental stages of *S. mekongi*, particularly the egg, cercaria, and adult female and male stages, remained largely uncharted until so far.

Our study aimed to address the knowledge gap, presenting a comprehensive analysis of miRNA profiles across different developmental stages of *S. mekongi* and providing insight into potential functional and evolutionary implications with other worm species.

## Results

To investigate the miRNA profiles across various life stages of *S. mekongi*, the pre-processed sequencing reads of small RNA (sRNA) were obtained with an average length ranging from 22 to 24 nt. Specifically, the reads averaged 24 nt for the egg stage, 23 nt for the cercaria stage, and 22 nt for both female and male *S. mekongi*. Furthermore, the average number of pre-processed sequencing sRNA reads were as follows: 2,322,436 reads for the egg stage, 598,628 reads for the cercarial stage, 328,958 reads for the female *S. mekongi*, and 297,212 reads for the male *S. mekongi* (Supplementary Table 1).

In an annotation of sRNA against the *S. japonicum* reference miRNA database across various life stages of *S. mekongi*, distinct numbers of miRNA reads were identified. For the egg stage, an average of 22,496 miRNA reads were detected. In contrast, the cercarial stage presented a markedly lower number, with an average of only 1772 annotated miRNA reads. Intriguingly, the female *S. mekongi* revealed an average of 1713 reads, whereas the male counterpart exhibited a significantly greater number with 10,365 reads on average (Supplementary Table 1).

**Figure 1:**
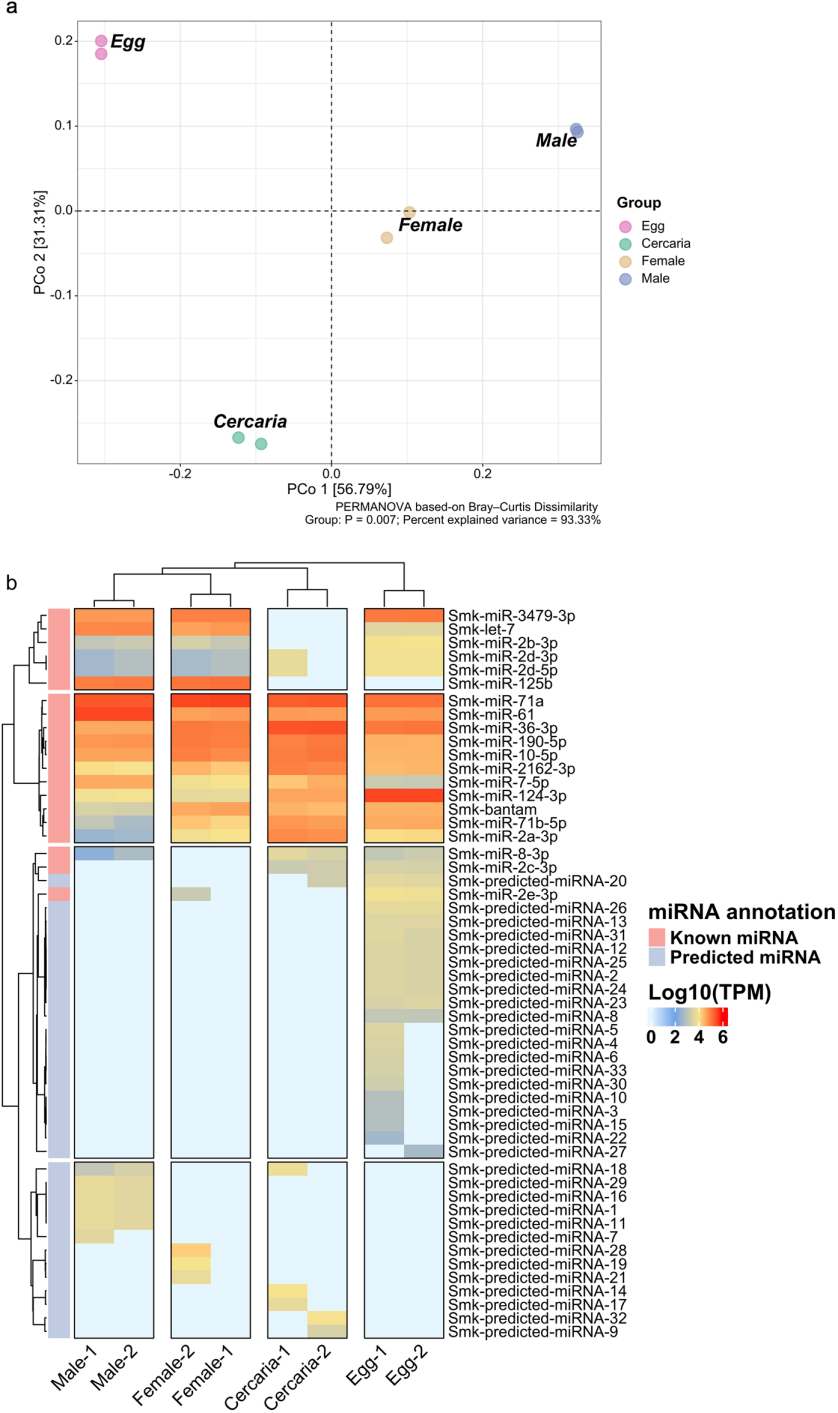
The miRNA profiling in *S. mekongi*. (**a**) Principal Coordinate Analysis (PCoA) of the *S. mekongi* miRNA in each stage was presented based on Bray–Curtis’s dissimilarity. Four distinct groups were observed, corresponding to Egg, Cercaria, Female, and Male. The first principal coordinate (PCo1) explained 56.79% of the variance, whereas the second principal coordinate (PCo2) accounted for 31.39% of the variance. A significant group differentiation was noted (PERMANOVA, P = 0.007), with a cumulative explained variance of 93.33%. (**b**) Unsupervised clustering analysis of the miRNAs from each stage of *S. mekongi* was conducted. The heatmap depicted the differential expression patterns of known and predicted miRNAs, as measured by Log10(TPM). The miRNA from each stage was clustered into distinct groups based on miRNA expression levels.

To address the variation in miRNA profiles of *S. mekongi* at each stage of life, PCoA was employed in conjunction with PERMANOVA at 1000 permutations, and unsupervised clustering was conducted. The PCoA revealed differences in miRNA profiles at each developmental stage. Notably, miRNA profiles of both female and male S. mekongi were more closely related to each other than to those of the egg or cercarial stages (Fig. 1a). In alignment with this observation, unsupervised clustering demonstrated the analogous clustering pattern, wherein the miRNA profiles of both female and male *S. mekongi* appeared in closer proximity to each other compared to the egg or cercarial stages (Fig. 1b).

Across the developmental stages of *S. mekongi*, miRNA profiles exhibit distinct variations, as demonstrated by both PCoA and unsupervised clustering. Notably, the miRNA profiles of the female and male stages are strikingly similar, suggesting shared genetic or developmental processes distinct from the egg and cercarial stages. Conversely, the early life stages (egg and cercarial) display unique miRNA signatures, reflecting their specialized molecular and developmental pathways.

To determine the biological roles of miRNAs at various stages of the schistosome, gene ontology (GO) analysis of miRNAs was conducted using annotated miRNA profiles of *S. japonicum* as a reference (Supplementary Figs. 1–4 and Supplementary Table 2)^15^.

In the egg stage of *S. mekongi*, certain miRNAs were identified as regulators of diverse biological processes, cellular components, and molecular functions, as illustrated in Supplementary Fig. 1. For biological processes, Smk-let-7 was found to associate with DNA repair, chromatin remodeling, histone acetylation, and intracellular signal transduction. Simultaneously, Smk-miR-36-3p was related to the regulation of DNA-templated transcription and RNA-mediated gene silencing, suggesting a potential role in the maintenance of stem cell populations within the schistosome developmental phase^16,17^. In terms of cellular components, Smk-let-7 was localized to the NuA4 histone acetyltransferase (HAT) complex and the nucleus, providing a clue for chromatin modification during developmental stages^18,19^. In contrast, Smk-miR-36-3p was associated with the CCR4-not Core Complex and the nucleus. As molecular functions, Smk-let-7 demonstrated DNA binding and protein kinase activities, while Smk-miR-2a-3p was shown specificity for histone binding and sequence-specific DNA binding.

During the cercarial stage of *S. mekongi*, Smk-miR-36-3p was identified as regulating transcription, DNA-templated regulation, mRNA processing, and RNA-mediated gene silencing, as shown in Supplementary Fig. 2. The role in the regulation of stem cell population maintenance was also predicted, suggesting broad implications in schistosome development^17^. Additionally, Smk-miR-71a and Smk-miR-2a-3p were associated with the regulation of transcription by RNA Polymerase II and DNA-templated transcription, respectively. For cellular components, Smk-miR-36-3p was localized to both the CCR4-not Core Complex and the nucleus. In molecular functions, Smk-miR-71a was linked with protein binding, while Smk-miR-2a-3p was associated with diverse functions, including histone binding, DNA-binding transcription factor activity, zinc ion binding, and sequence-specific DNA binding.

The miRNA functions in both female and male *S. mekongi* were similar, as depicted in Supplementary Figs. 3 and 4. In the biological process, miRNAs such as Smk-miR-71a, Smk-miR-36-3p, Smk-miR-2a-3p, and Smk-let-7 were consistently identified in both genders. These miRNAs were implicated in processes including transcription regulation by RNA polymerase II, DNA-templated regulation, mRNA processing, and RNA-mediated gene silencing. Notably, Smk-let-7 was associated with DNA repair, chromatin remodeling, and intracellular signal transduction. The association of Smk-miR-36-3p with stem cell population maintenance was predicted, suggesting potential roles in reproductive processes^17,20^. In terms of cellular components and molecular function, miRNAs in both genders were localized to specific cellular structures and revealed different molecular functions. For example, Smk-let-7 was related to the NuA4 HAT complex and the nucleus.

However, the miRNA profile of male *S. mekongi* revealed several predicted miRNAs, including Smk-predicted-29, Smk-predicted-16, Smk-predicted-11, and Smk-predicted-1. These miRNAs were mainly associated with transcription by RNA Polymerase II and microtubule-based movement, with primary molecular activities characterized by protein binding, suggesting roles in molecular transport and intracellular dynamics specific to the mature male stage of *S. mekongi*^21,22^. Further studies are crucial to determine the exact functions of these predicted miRNAs.

**Figure 2:**
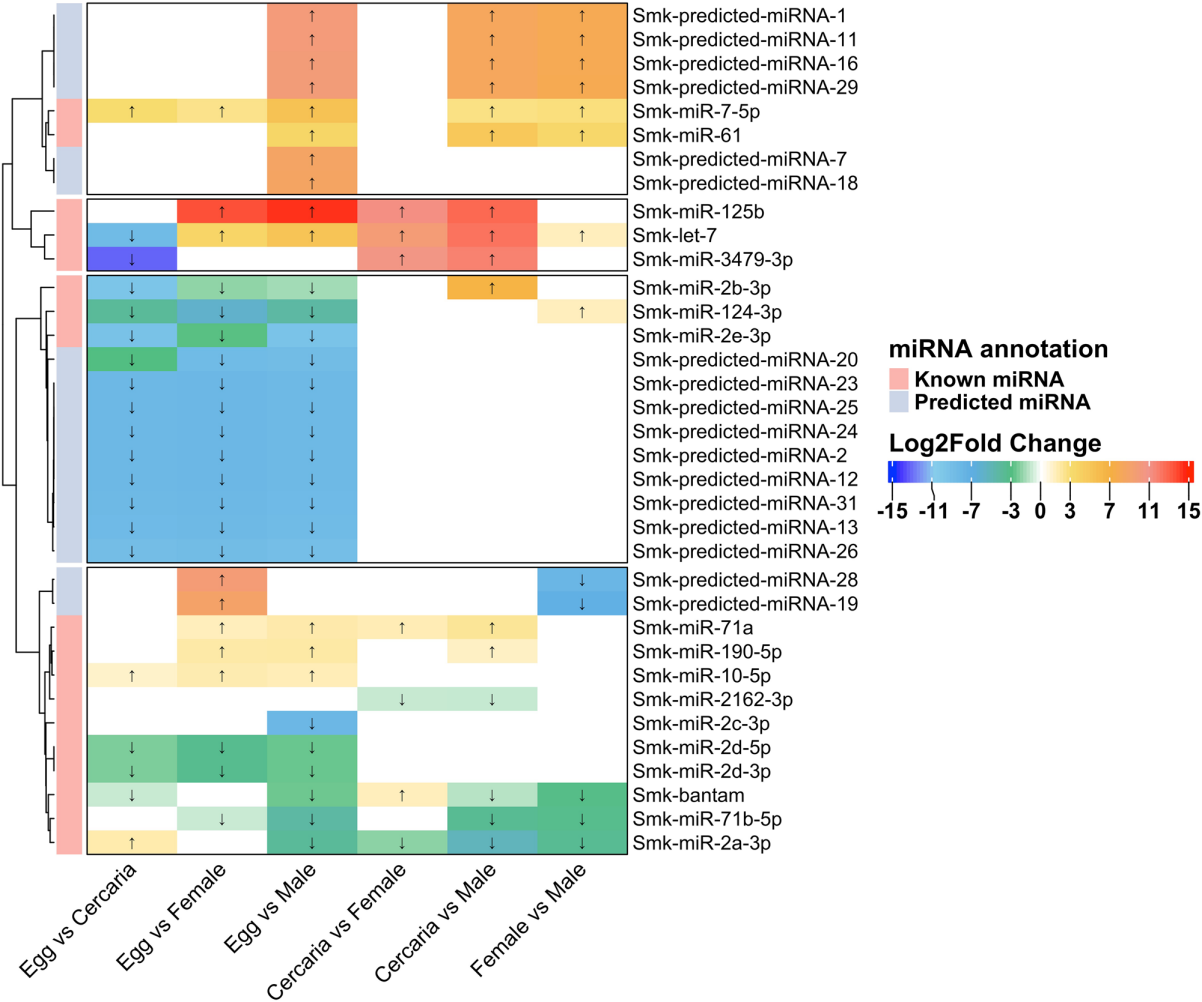
Differential expression analysis of miRNAs across developmental stages in *S. mekongi*. The heatmap displayed the LCF in miRNA expression between the stages, as indicated by the color gradient from blue (downregulated) to red (upregulated). Several notable expression patterns were observed. For instance, specific miRNAs exhibited marked upregulation in the egg compared to cercaria, while others showed pronounced downregulation in the egg compared to male. The arrows on the heatmap indicated the direction of fold change for the respective miRNAs. The miRNAs depicted in the heatmap were determined to be of statistical significance, with an FDR of < 0.05.

Differential expression analyses of miRNAs were performed across developmental stages of *S. mekongi*, including the egg, cercarial, female, and male stages (Fig. 2). The expression of miRNAs in the egg stage, when compared to the cercarial stage, revealed a marked downregulation for several miRNAs. The most pronounced was Smk-miR-3479-3p, which exhibited a Log2FoldChange (LFC) of − 13.45. This was followed closely by Smk-miR-2b-3p and Smk-miR-2e-3p, which registered values of − 9.90 LFC and − 9.55 LFC, respectively. Additionally, miRNAs such as Smk-predicted-26, Smk-predicted-20, and Smk-predicted-13 showed significant downregulation within a range of − 8.33 to − 8.95 LFC. Interestingly, Smk-let-7 was upregulated in the egg stage relative to the cercarial stage, showing an LFC of − 8.67.

When comparing the egg stage to the female *S. mekongi*, Smk-miR-125b was revealed as the most upregulated miRNA, with an LFC of 13.65. Predicted miRNAs exhibited upregulation included Smk-predicted-28 and Smk-predicted-19, which displayed values of 9.81 and 9.04 LFC, respectively. On the contrary, miRNAs such as Smk-miR-2e-3p, Smk-miR-124-3p, and Smk-miR-2d-3p were observed to be downregulated, with LFC ranging from − 2.01 to − 6.00.

In the comparison between the egg stage and male *S. mekongi*, Smk-miR-125b was consistently dominated in terms of upregulation with an LFC of 14.44. Certain predicted miRNAs (i.e., Smk-predicted-29, Smk-predicted-16, Smk-predicted-11, and Smk-predicted-1) showed significant upregulation, each with values hovering around 9.93 or 9.94 LFC. On the downregulation, Smk-miR-2e-3p was presented a LFC of − 9.67, while Smk-miR-2b-3p and Smk-miR-2c-3p were shown changes of − 1.71 LFC and − 8.20 LFC, respectively.

To compare the cercarial stage with female and male *S. mekongi*, most miRNAs displayed consistent expression across the stages. Notably, Smk-miR-3479-3p, Smk-let-7, and Smk-miR-125b were significantly upregulated in both comparisons, ranging from 9 to 12.80 LFC. Moreover, Smk-miR-2a-3p showed downregulation in both stage comparisons, with LFC of − 2.12 and − 5.37 in female and male, respectively. However, predicted miRNAs (i.e., Smk-predicted-29, Smk-predicted-16, Smk-predicted-11, and Smk-predicted-1) were shown upregulation in only male *S. mekongi* but not in female.

As the comparison of female and male *S. mekongi*, Smk-let-7 revealed a downregulation with a LFC of − 1.39. Moreover, predicted miRNAs, including Smk-predicted-29, Smk-predicted-16, Smk-predicted-11, and Smk-predicted-1, were observed to be downregulated with an LFC of − 7.86.

To understand the roles of differentially expressed miRNAs at various developmental stages of *S. mekongi*, a GO analysis was conducted to discern the biological process associated with these miRNAs (Supplementary Fig. 5). In considering the miRNA biological processes in both egg and cercarial stages, it was observed that these biological processes played a pivotal role in cellular migration and neurological development. This was substantiated by the in-silico identification of processes such as substrate-dependent cell migration, regulation of establishment of planar polarity, and neuron projection extension (Supplementary Fig. 5a). These results may suggest that the schistosome might undergo intricate cellular and neurological adaptations in preparation for transition to the free-swimming, infectious cercarial stage^23,24^.

Focusing on the egg stage and female *S. mekongi*, the biological processes were shifted towards cellular signaling and molecular synthesis, with protein kinase B signaling, positive regulation of translational initiation, and mRNA polyadenylation being prominent (Supplementary Fig. 5b). These processes may indicate the biological processes focus on cellular growth, energy metabolism, and protein synthesis, reflecting the preparation for reproductive maturity within the definite host^25,26^.

In the comparison between the egg stage and male *S. mekongi*, the processes associated with cellular migration, fibroblast migration, and microtubule-based transport were predominant, implying the physiological and morphological transformations required for the development of male-specific structures and mating (Supplementary Fig. 5c).

Interestingly, the transition from the cercarial stage to both the female and male stages revealed a high degree of similarity in altered biological processes, including but not limited to protein kinase B signaling, positive regulation of translational initiation, and TOR signaling (Supplementary Fig. 5d and e). This parallelism in biological processes may imply a shared adaptive response in cellular architecture, signaling pathways, and molecular synthesis as the parasite develops within the definite host, irrespective of the gender^21^.

The exploration of BP in the female compared to male *S. mekongi* revealed a continuation of shared processes such as microtubule-based transport and protein kinase B signaling, suggesting the inherent sexual dimorphism while underscoring shared metabolic or structural pathways integral to the survival of both sexes within the host (Supplementary Fig. 5f).

**Figure 3:**
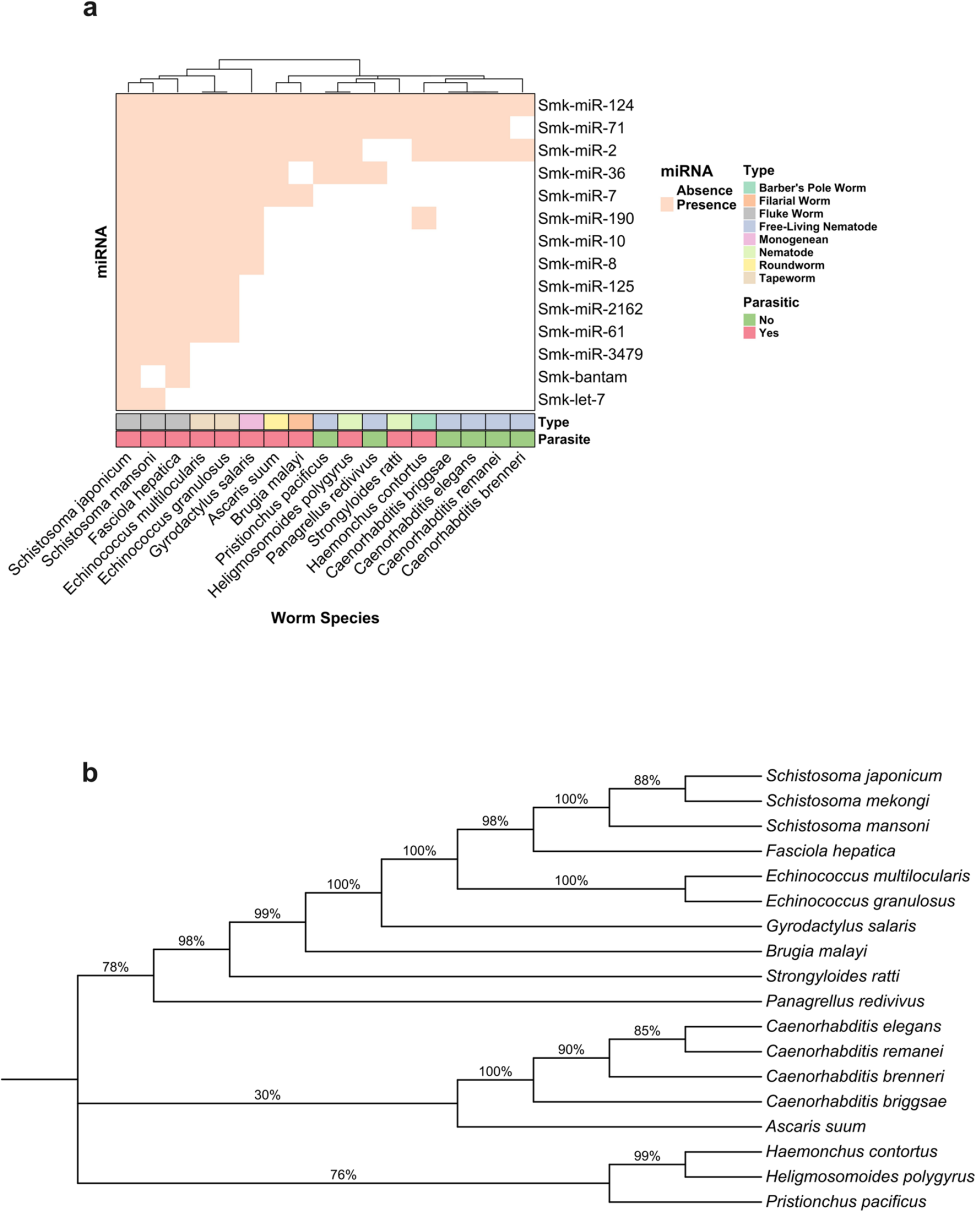
Orthologous miRNA and phylogenetic analysis in *S. mekongi* across various worm species. Orthologous miRNA patterns across diverse worm species were explored using. (**a**) The heatmap showed the presence (indicated by a filled square) or absence (blank square) of miRNAs across the examined worm species. Each row corresponds to a specific miRNA from *S. mekongi*, while columns represent the different worm species. The species were classified into various types, such as barber’s pole worm, filarial worm, free-living nematode, monogenean, nematode, roundworm, and tapeworm, as highlighted by the color bar beneath the heatmap. Additionally, a distinction was made between parasitic and non-parasitic species. Several miRNAs, including Smk-miR-124 and Smk-miR-71, were observed to have broad distribution across multiple worm species. In contrast, certain miRNAs, such as Smk-bantam and Smk-let-7, revealed a more restricted presence among species. (**b**) The phylogenetic analysis was constructed using COX1 protein sequences of various worm species, revealing the consistency of evolutionary relationship among worms.

In the orthologous miRNA analysis conducted for *S. mekongi* against various worm species, distinctive presence-absence patterns of miRNAs were observed, offering valuable insights into the evolutionary relationships and potential functional roles of these miRNAs (Fig. 3 and Supplementary Table 3).

Among the Fluke worms, it was notable that *S. japonicum*, *S. mansoni*, and *Fasciola hepatica* exhibited a high degree of miRNA conservation. This observation suggested a potential shared evolutionary history or similar functional roles of these miRNAs in the biology and parasitism of fluke worms. The consistent absence of Smk-miR-2 and Smk-bantam might indicate a lineage-specific loss or functional redundancy in these species.

The free-living nematodes, despite their non-parasitic mode of living, shared a unique miRNA profile distinct from parasitic worms. The consistent absence of miRNAs across these free-living worms might reflect the complex interplay of evolutionary, functional, and environmental factors that shape the miRNA landscape of these organisms.

The tapeworms, *Echinococcus multilocularis* and *E. granulosus*, exhibited a high degree of miRNA conservation, implying potential shared evolutionary trajectories or functional roles. The absence of bantam in these species could indicate a non-crucial role in tapeworms or a lineage-specific loss.

*Nematodes* presented an intriguing pattern. Their miRNA profiles varied significantly, suggesting diverse evolutionary paths or adaptations to different host environments. The presence of Smk-miR-2 in some and the absence in others might indicate a variable role across nematode species.

Interestingly, the monogenean *Gyrodactylus salaris* displayed a miRNA profile distinct from fluke worms and free-living nematodes. This distinction provided evidence for the unique evolutionary lineage or adaptation strategies.

Upon comparing the miRNA profile of *S. mekongi* with these species, it was evident that the conservation or divergence of certain miRNAs might be intricately linked to their ecological niches, host interactions, or evolutionary trajectories. This analysis was instrumental in understanding the evolutionary significance of these miRNAs across various worm species.

## Discussions

In the present study, we explored the miRNA profiles across various developmental stages of the *S. mekongi* schistosome, providing a comprehensive understanding of their potential roles and evolutionary significance, in which these aspects have never been studied in this species.

The combined use of PCoA with PERMANOVA and unsupervised clustering provided a robust framework to dissect the intricate variations in miRNA expression across *S. mekongi* developmental stages. PCoA effectively transformed the multidimensional miRNA data, elucidating clear patterns of similarities and differences between stages, particularly highlighting the pronounced similarity in miRNA profiles between female and male *S. mekongi*. This observation may imply potential shared genetic or developmental trajectories distinct from early life stages^13,14,16^.

A striking observation from our analysis was the similarity in miRNA profiles between female and male *S. mekongi*. This close association in miRNA profiles between genders suggests potential shared post-transcriptional regulation or developmental processes, which, perhaps, are distinct from the processes driving the early life stages, such as the egg and cercarial stages^12,18,27^. These findings highlight that, after the cercarial stage, the schistosome experiences a significant change in environment, leading to greater similarity in the developmental paths of both male and female *S. mekongi*^12,27^.

The differential expression of certain miRNAs, notably Smk-miR-3479-3p, Smk-miR-2b-3p, and Smk-miR-2e-3p, across stages warrants further exploration. Their pronounced downregulation in the egg stage compared to the cercarial stage suggests they may play significant roles in the early developmental processes^16,27,28^. On the other hand, the upregulation of Smk-let-7 relative to the cercarial stage may imply a potential regulatory role in cellular differentiation^18,19^.

Furthermore, the in-silico identification of predicted miRNAs in male *S. mekongi* offers a promising avenue for future research. The association of these predicted miRNAs with specific biological processes, such as RNA polymerase II-mediated transcription and microtubule-based movement, provided an insight into potential involvement in male-specific developmental or reproductive processes^22,29^. However, a more in-depth functional analysis is necessary to elucidate their exact roles and significance in gender-specific development.

The orthologous miRNA analysis conducted on *S. mekongi*, compared with various worm species, revealed valuable insights into the living modes and evolutionary relationships of these worms. This was achieved through unsupervised clustering based on the presence or absence of miRNAs, as represented in a heatmap (Fig. 3a). This is consistent with the phylogenetic relationships among worm species, which were established by cytochrome c oxidase I (COX1) protein sequences (Fig. 3b).

**Figure 4:**
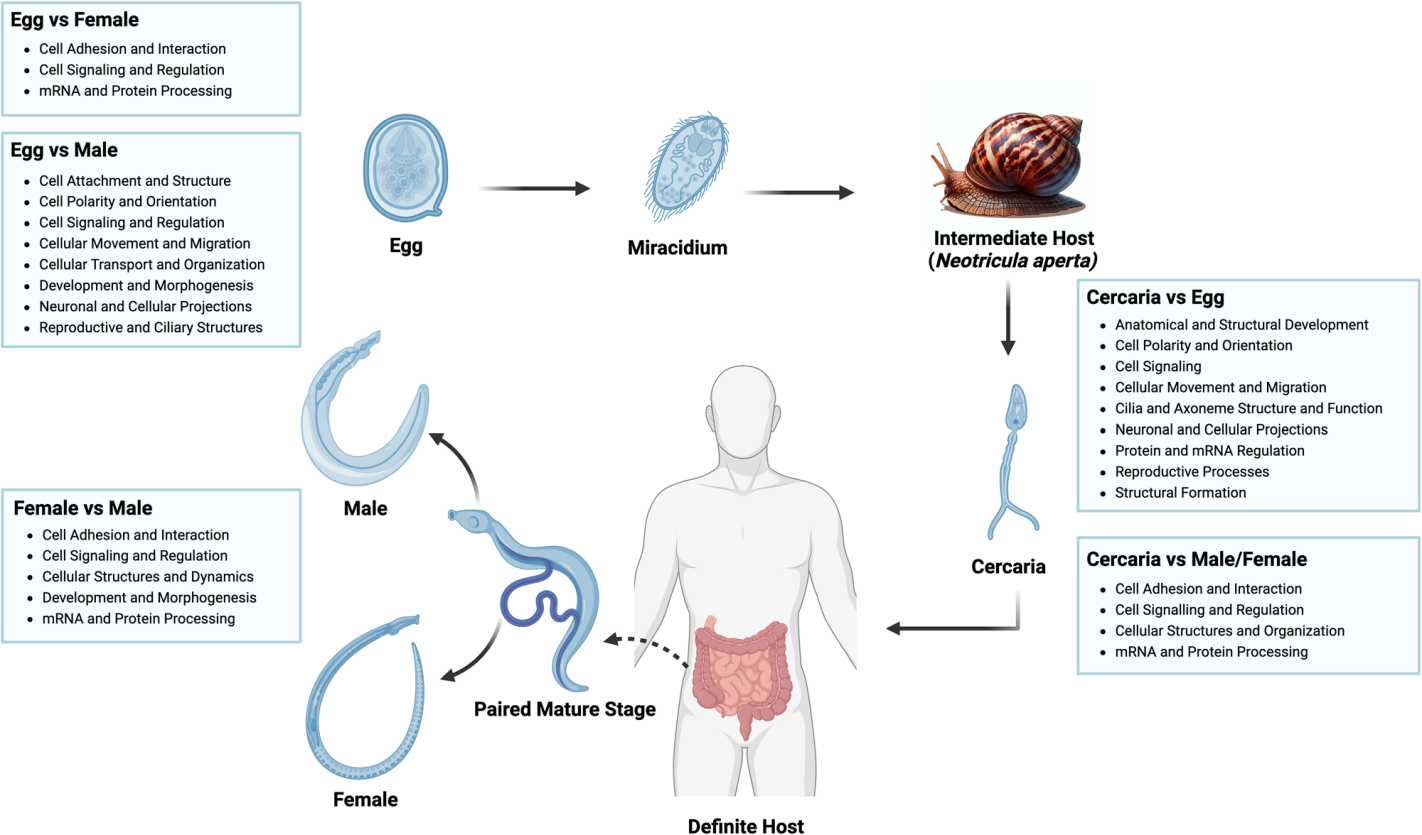
Graphical summary of biological processes in *S. mekongi* in each stage of life. The lifecycle stages of *S. mekongi* were depicted, including the egg, cercaria, female, and male stages, and associated with the biological processes of miRNA profiles in stage-wise comparison (This image was created with BioRender.com).

The intricate analysis of the miRNA profile across different developmental stages of *S. mekongi* has allowed for a profound comprehension of the regulation in various biological processes pivotal to the schistosome life cycle. By juxtaposing the miRNA expression across the stages, crucial facets of *S. mekongi* physiology and developmental trajectory were unraveled (Fig. 4).

Beginning with the foundational egg stage, biological processes such as cellular adhesion and interaction, cellular signaling and regulation, and mRNA and protein processing were at the forefront. While the apparent dormancy egg stage might suggest a state of inactivity, these processes underline an engagement in vital cell-to-cell interactions, possibly in anticipation of hatching and progression to the miracidium phase or schistosome-host interaction^16,30^. This active participation in cellular signaling and mRNA processing across both the egg and female stages may suggest the essence of gene regulation and protein synthesis in facilitating the reproductive processes^31,32^.

When contrasting the egg stage with the male stage, there was an expansive range of biological processes in play. Of note, the processes associated with cellular movement and migration were revealed. This provides a clue at the potential possession of unique cellular structures or mechanisms tailored for movement in male *S. mekongi*, possibly to locate and synchronize with the female^33,34^. Furthermore, the emphasis on developmental and morphogenesis processes, coupled with neuronal and cellular projections, provides insights into the structural and functional differentiation of male vis-à-vis the egg stage^16,29^.

The cercaria stage, a free-swimming infectious larval phase, exhibited key biological process regulations focused on anatomical development, cell polarity, and cilia and axoneme structures. These processes may indicate the adaptability of cercaria to aquatic environments, optimized for mobility and infectivity^35,36^. Emphasizing cellular movement and migration provides clues on molecular readiness for host-seeking and successful infection, indicating a refined adaptation for transitioning through the aquatic phase to the definitive host^35,36^.

Upon closer examination of the female and male *S. mekongi*, several parallels with the egg stage comparison to female *S. mekongi* became evident. This similarity may suggest conservation of certain cellular and molecular attributes from the egg stage to the adult phases in *S. mekongi*^13,16,34^. However, the distinct processes observed, such as variations in cellular adhesion and interaction, cellular signaling and regulation, and mRNA and protein processing, highlight the nuanced differences between the male and female schistosomes. These differences, while subtle, provide a clue on the specialized roles or adaptations each gender might have developed to fulfill the respective roles within the host or during the reproductive cycle ^13,20^.

Comparative miRNA profiling with other worm species further enriched our understanding of the evolutionary and functional relevance of miRNAs in *S. mekongi*. The high degree of miRNA conservation observed among fluke worms, including *S. japonicum*, *S. mansoni*, and *F. hepatica*, suggests potential shared evolutionary histories or functional roles of these miRNAs in fluke worm parasitism. On the other hand, the unique miRNA profiles in free-living nematodes, tapeworms, and the monogenean *G. salaris* indicate lineage-specific adaptations or evolutionary trajectories^37–39^.

While several miRNAs from each life stage of *S. mekongi* were identified through manual curation against databases, the function of many remains unclear due to database limitations. Our study also faced constraints in validating results with absolute quantitative techniques, such as qPCR and profiling miRNAs in miracidia and sporocyst stages. Nevertheless, this preliminary investigation establishes a foundation for subsequent research into the functional significance of specific miRNAs in *S. mekongi* and their potential application as therapeutic targets for schistosomiasis.

For instance, stage-specific miRNAs of *S. japonicum*, such as Sja-miR-36, Sja-miR-71 and Sja-bantam, play crucial roles in parasite development and disease progression^12,40,41^. These miRNAs, notably found in host plasma, were associated to schistosomiasis-related cancers, particularly bladder and liver cancers^12,40,41^. Additionally, host miRNAs such as miR-223 and miR-454 were observed to contribute to liver pathology during infection by influencing key signaling pathways (i.e., MAPK and TGF-β)^12,40,41^.

Thus, future perspectives should aim to understand the mechanisms of host-parasite interactions and miRNA-mediated pathology, in order to effectively utilize miRNAs in schistosomiasis monitoring. This understanding is crucial within the context of host-parasite dynamics and could pave the way for disease prognosis.

In conclusion, the present study conducted an examination of miRNA profiles across various developmental stages of *S. mekongi*, offering insights into the potential developmental roles of miRNA.

## Methods

### Sample collection

This study was conducted with approval from the Faculty of Tropical Medicine Animal Care and Use Committee, Mahidol University (Approval No. FTM-ACUC 015/2021), adhering to ARRIVE guidelines and relevant regulations. We utilized a laboratory strain of *S. mekongi*, maintained in *Neotricula aperta* snails and ICR mice at Mahidol University’s Applied Malacology Laboratory. Snails, originating from the Mekong River, were cultured in the laboratory. For the experiment, we procured ten 8 week-old female ICR mice from the National Laboratory Animal Center. Post 8 week infection, mice were euthanized using compressed CO_2_, and *S. mekongi* adults were extracted via hepatic perfusion with 0.85% saline. The liver and intestines were then processed to separate parasite eggs, using sieves of various sizes (425, 250, 106, and 45 µm). Additionally, cercariae were collected from 8 week infected snails after 2 h of light exposure, observed under a stereomicroscope. This study used approximately 3000 eggs, 100 cercariae, and 20 adult worms (10 males and 10 females) for further processes.

### RNA extraction

The total RNA was extracted from samples (egg, cercaia, and adult parasites). All samples were lysed by lysis buffer and homogenized by TissueLyser LT (Qiagen, Germany) at 50 Hz for 5 min using RNeasy Mini Kit (Qiagen, Germany) following the manufacture’s instruction. RNA concentrations were measured using NanoPhotometer^®^ C40 (Implen, Germany) and Qubit RNA HS assay kit (Invitrogen, USA). To ensure the quality of RNA before proceeding with library preparation and sequencing, the integrity and concentration of RNA were assessed using a Bioanalyzer. Only RNA samples that were passed the quality criteria specified by Vishuo Biomedical Pte., Ltd. for small RNA sequencing were used.

### Small RNA (sRNA) sequencing and bioinformatic analysis

The library preparation and sRNA sequencing were performed commercially by Vishuo Biomedical Pte., Ltd., according to the manufacturer’s standard protocols. The library preparation was performed using a TruSeq Small RNA Library Prep Kit for Illumina^®^ (Illumina, USA). Briefly, 10 ng of sRNA was used for library preparation. The 3′´SR Adaptor was ligated to the sRNA using 3′´Ligation Enzyme. The excess of 3′´SR Adaptor was hybrid with SR RT Primer for Illumina to prevent adaptor-dimer. Then, 5′SR Adaptor was ligated to the sRNA using 5′´Ligation Enzyme. Next, the first strand cDNA was synthesized using ProtoScript^®^ II Reverse Transcriptase (New England Biolabs, USA). Each sample was then amplified by PCR using TruSeq Small RNA Indices primers; the PCR products were purified by DNA clean beads. The purified products of 140–160 bp were recovered and cleaned up using PAGE, and then validated using an Agilent 2100 Bioanalyzer (Agilent Technologies, USA). Then, libraries with different indexes were multiplexed. The qualified libraries were sequenced paired end (PE150) sequencing on the NovaSeq 6000 System instrument (Illumina, USA) according to the manufacturer’s protocols.

Raw Fastq datasets were performed pre-processing steps, in which sequencing adapters were removed, quality was trimmed (Q < 20) and reads shorter than 18 bp were discarded using Trimmomatic (v0.30)^42^. Following pre-processing, Smk-miRNA identification was carried out using miRDeep2^43^. The pre-miRNAs of predicted miRNAs were validated by observing their minimum Gibbs free energy (MFE) and secondary hairpin structure using RNAfold in the ViennaRNA Package 2.0^44^. To be considered potential miRNAs, the pre-miRNAs had to exhibit an MFE lower than − 5.0 kcal/mol and demonstrate the characteristic topology of pre-miRNAs (Supplementary Table 4)^45,46^.

Differential expression analysis was then conducted using DESeq2^47^, with miRNAs showing significant differential expression defined by Benjamini and Hochberg’s adjusted P-value (False Discovery Rate; FDR) < 0.05.

Gene ontology (GO) analysis was conducted using GOSeq (v1.34.1)^48^, and significance was set at Benjamini and Hochberg’s FDR < 0.05. In-silico prediction of miRNA-mRNA target interactions was performed using Miranda^49^ and manually curated against the *S. japonicum* miRNA database (https://wormbase.org), which is evolutionarily closely related to *S. mekongi* ^50,51^. The GO network analysis of the identified miRNAs was performed using Cytoscape^52^.

For phylogenetic analysis as depicted in Fig. 3b, the COX1 protein sequences of each worm species shown in Fig. 3a were aligned with MUSCLE (version 5.0)^53^ and constructed the tree with FastTree (version 2.1)^54^.

The PCoA was performed with PERMANOVA (at 1000 permutations) and Bray–Curtis dissimilarity using the Vegan R package^55^. Unsupervised clustering was conducted with the ComplexHeatmap R package^56^.

### Ethical approval

All procedures were approved by the Faculty of Tropical Medicine Animal Care and Use Committee (FTM-ACUC), Mahidol University (Approval number: FTM-ACUC 015/2021).

### Supplementary Information

Supplementary Figures.

Supplementary Table 1.

Supplementary Table 2.

Supplementary Table 3.

Supplementary Table 4.

## Data Availability

The sequencing data supporting the findings of this study was deposited with NCBI-GenBank (accession numbers PRJNA1029332).
