# Supplementary material for: Comprehensive analysis of miRNA profiling in Schistosoma mekongi across life cycle stages
Source: Sci Rep. 2024 Jan 29;14:2347. doi: 10.1038/s41598-024-52835-5 (PMC10822868; doi:10.1038/s41598-024-52835-5)
Supplement: Supplementary file 1 — Supplementary Figures. [file 41598_2024_52835_MOESM1_ESM.docx]

**
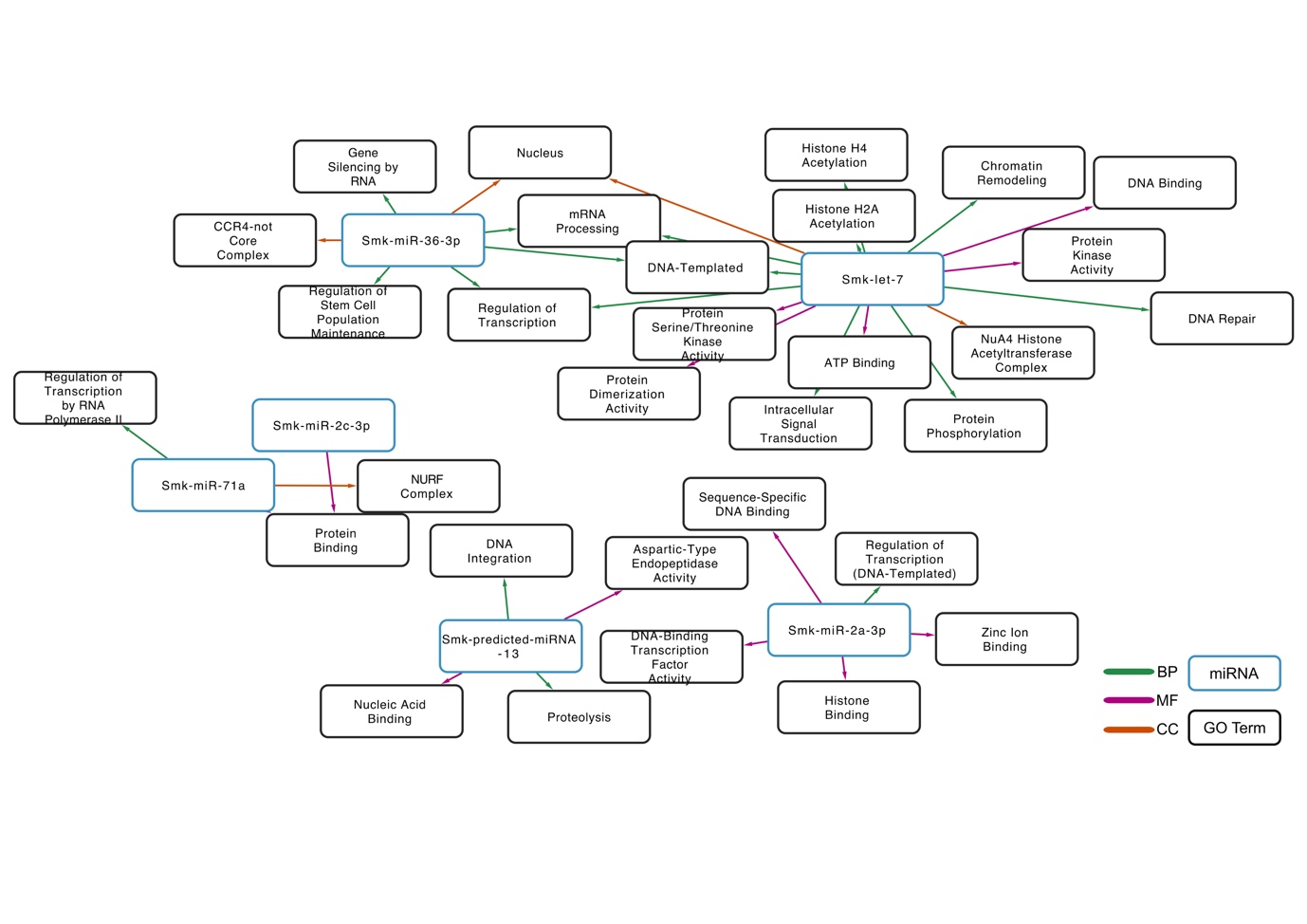
Supplementary Information**

**Supplementary Figure 1.** Gene Ontology (GO) analysis of miRNA profiling in the *S. mekongi* egg stage. The interconnected nodes were represented the association of specific miRNAs (blue) with their corresponding biological processes (BP, green), molecular functions (MF, magenta), and cellular components (CC, orange).

**
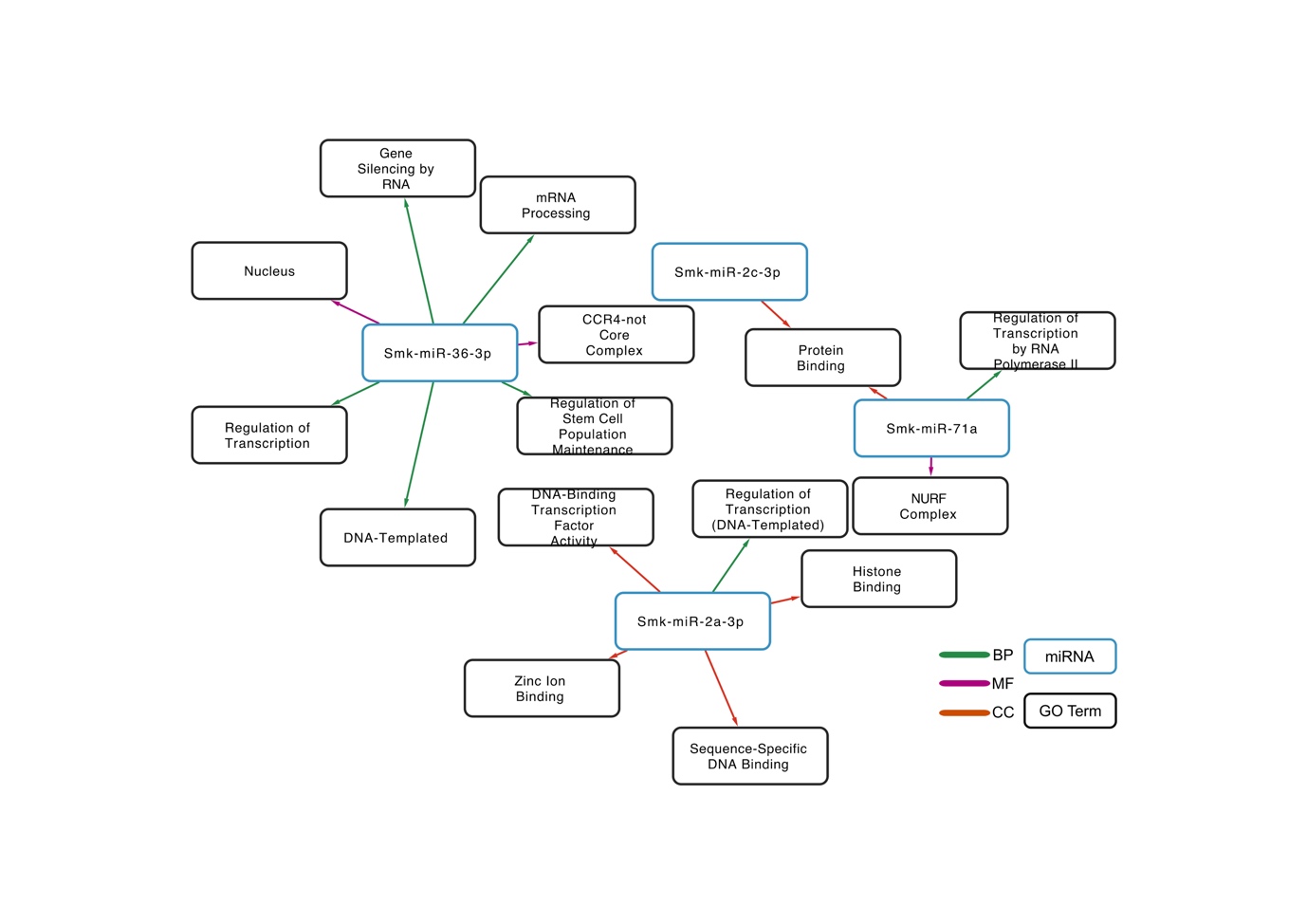
Supplementary Figure 2.** Gene Ontology (GO) analysis of miRNA profiling in the *S. mekongi* cercarial stage. The interconnected nodes were represented the association of specific miRNAs (blue) with their corresponding biological processes (BP, green), molecular functions (MF, magenta), and cellular components (CC, orange).

**
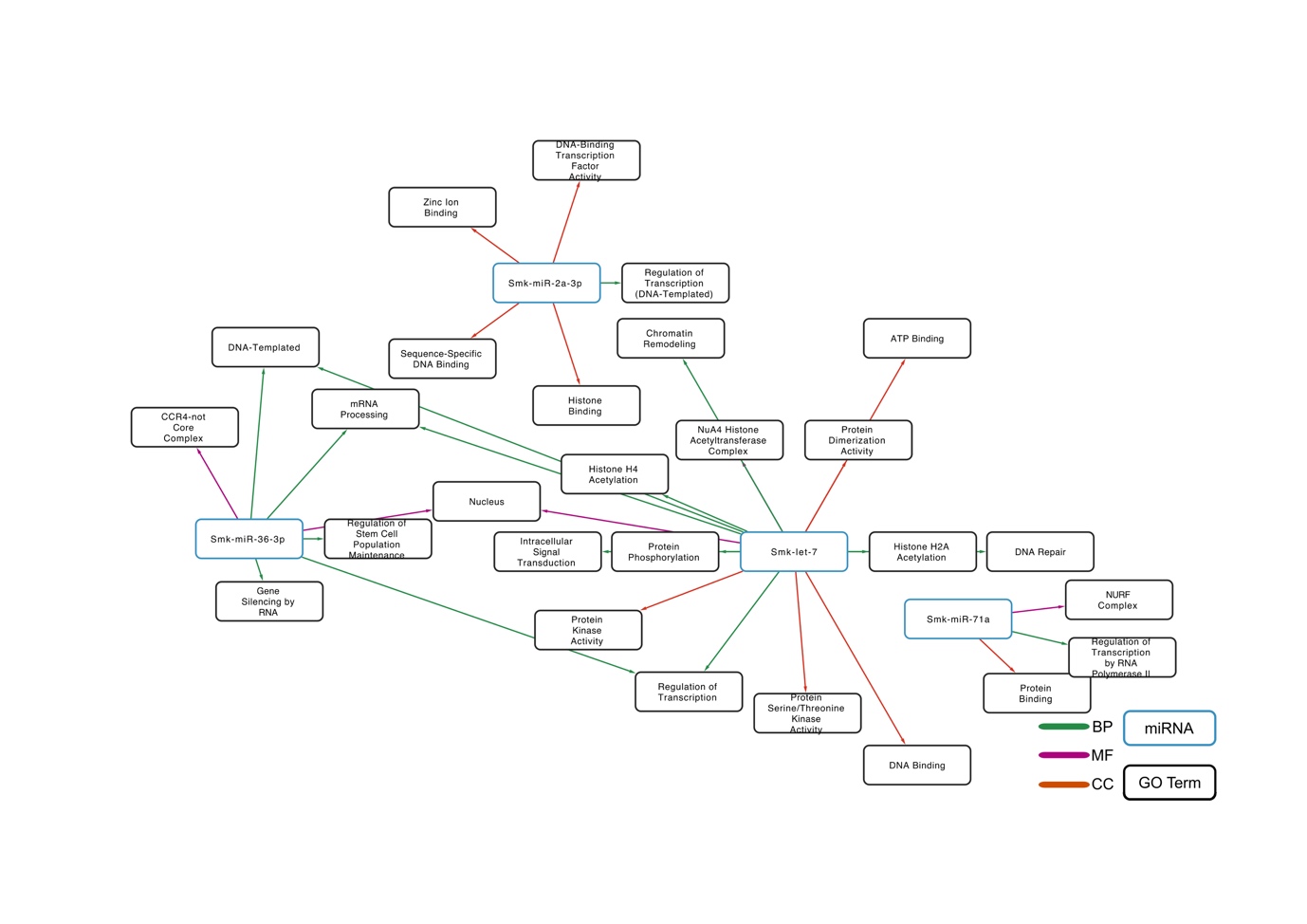
Supplementary Figure 3.** Gene Ontology (GO) analysis of miRNA profiling in the female *S. mekongi*. The interconnected nodes were represented the association of specific miRNAs (blue) with their corresponding biological processes (BP, green), molecular functions (MF, magenta), and cellular components (CC, orange).

**
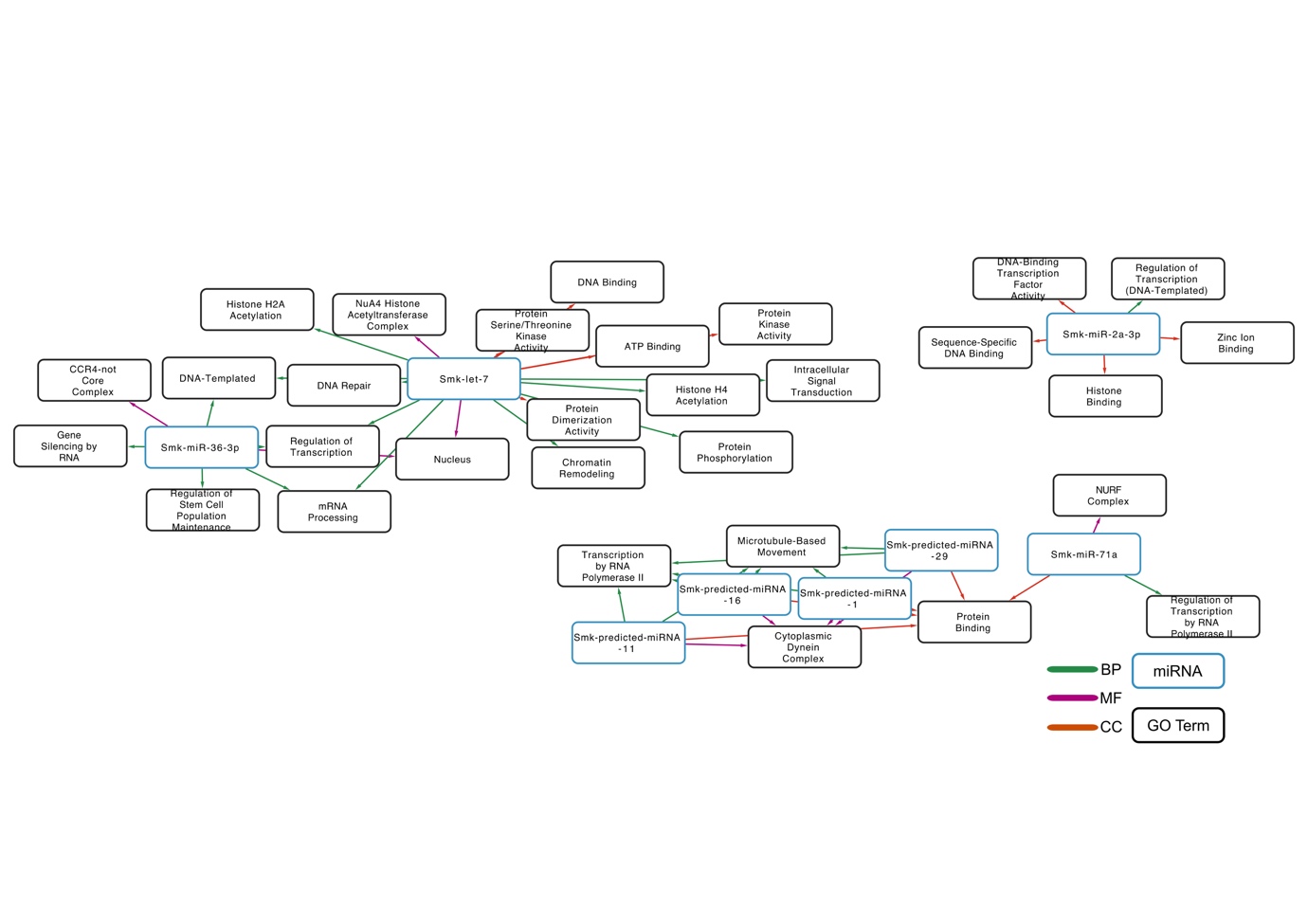
Supplementary Figure 4.** Gene Ontology (GO) analysis of miRNA profiling in the male *S. mekongi*. The interconnected nodes were represented the association of specific miRNAs (blue) with their corresponding biological processes (BP, green), molecular functions (MF, magenta), and cellular components (CC, orange).

**
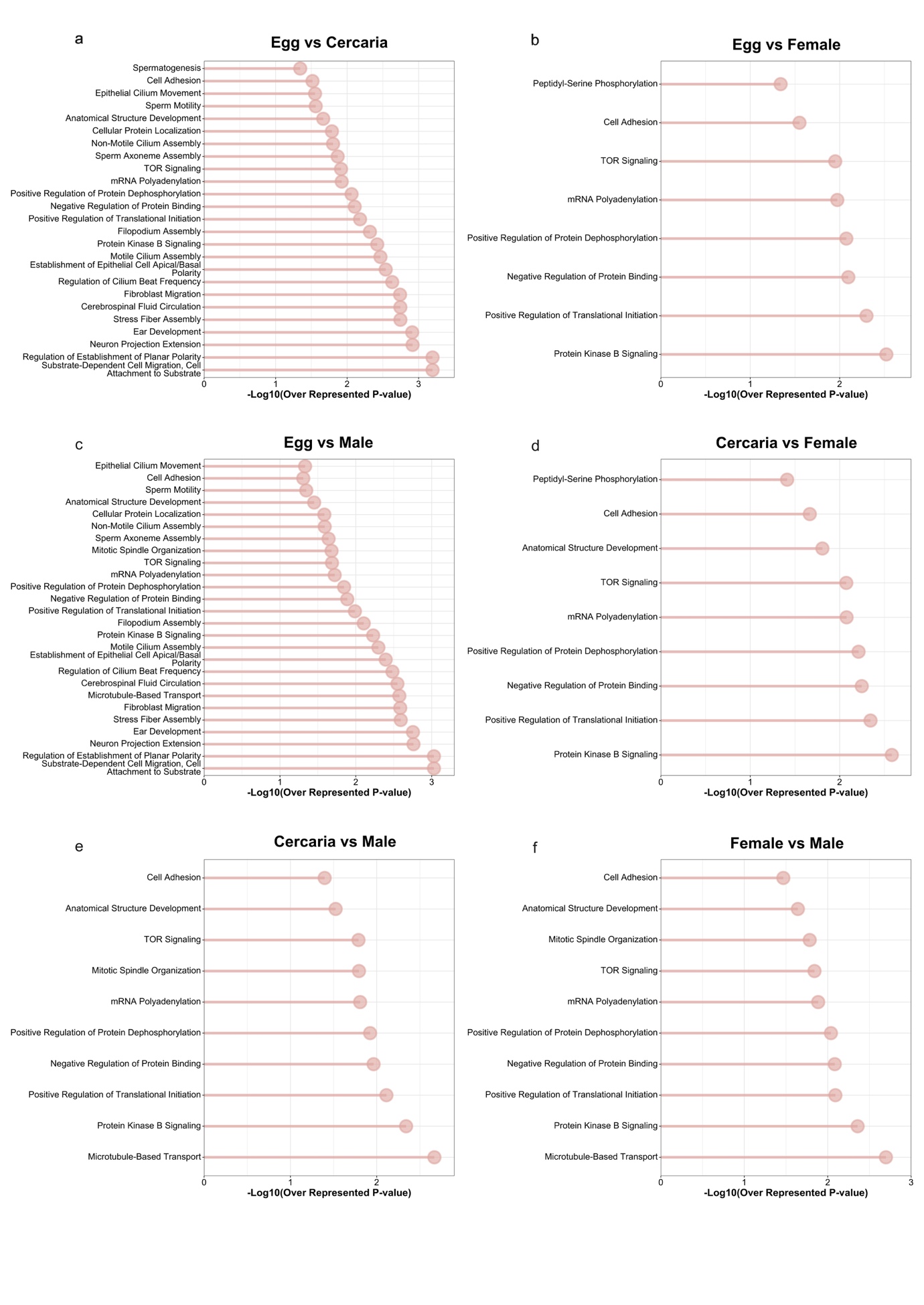
Supplementary Figure 5.** Biological Process comparison of differentially expressed miRNA across developmental stages of *S. mekongi*. (**a**) Egg and cercarial stage comparison was revealed processes ranging from spermatogenesis to regulation of planar polarity establishment. (**b**) Egg and female stage comparison was showed involvement in pathways such as peptidyl-serine phosphorylation and protein kinase B signaling. (**c**) Egg and male comparison was demonstrated biological processes including epithelial cilium movement and neuron part extension. (**d**) Cercaria and female comparison suggested biological processes such as anatomical structure development and mRNA polyadenylation. (**e**) Cercarial stage and male comparison was revealed biological processes such as microtubule-based transport and protein kinase B signaling. (**f**) Female and male comparison was revealed biological processes such as mitotic spindle organization and negative regulation of protein binding.

**Supplementary Figure 6.** Phylogenetic relationships of worm species based on cytochrome c oxidase I (COX1) protein sequences. The phylogenetic tree illustrates evolutionary relationships among selected worm species based on the COX1.
